# Factors influencing Australian chiropractors who choose not to join national professional associations: a qualitative study

**DOI:** 10.1186/s12998-020-00351-4

**Published:** 2020-12-01

**Authors:** Stanley I. Innes, Vicki Cope, Kenneth J. Young

**Affiliations:** 1 College of Science, Health, Engineering and Education, Murdoch University, Murdoch, Australia; 2 School of Sport and Health Sciences, University of Central Lancashire, Lancashire, England

**Keywords:** Chiropractic, Professional associations, Qualitative research, Barriers, Facilitators

## Abstract

**Background:**

Professional associations (PAs) are perceived to promote their professions and support their members. Despite these advantages, about 1 in 3 Australian chiropractors choose not to belong to either of the two PAs. Our study had two objectives: 1) to explore the views of non-member chiropractors about PAs in general; 2) seek to understand the motivations of non-member Australian chiropractors about not joining a PA.

**Methods:**

This qualitative descriptive study utilised in-depth semi-structured interviews with open-ended questions for thematic analysis and was conducted from January to April 2020. Nine participants were interviewed before no new themes were articulated. Participants had to be registered chiropractors who had not been members of a PA for at least three years. Recruitment was through a Facebook advertisement and snowball sampling. Interviews were transcribed and imported into NVivo qualitative analysis software, allowing identification of key concepts surrounding non-membership of chiropractic PAs.

**Results:**

Five themes were identified. 1) A tarnished image, suggested the profession has a poor standing in the eyes of the public and other health professionals. 2) Not worth the money, expressed the annual membership dues were not viewed as good value for money. 3) Going it alone / what’s in it for me? indicated there was no direct benefit or anything deemed essential for practice. 4) Two warring factions, reflected not wanting to be seen to be part of the internal conflict between conservative and evidence-based practitioners. 5) Lack of visibility, described no visible presence or strong communication that clearly displayed the advantages of membership.

**Conclusions:**

Non-members are looking for PAs to enhance the respectability of the profession in a manner that ultimately results in increased patient volume and the provision of readily accessible day-to-day resources and information. These results can inform the construction of a survey for the broader chiropractic non-membership community to confirm and expand upon these findings and potentially improve PAs.

**Supplementary Information:**

The online version contains supplementary material available at 10.1186/s12998-020-00351-4.

## Background

Professional associations (PAs) are perceived to play an important role in supporting their membership and improving the quality of the members working lives [1–3]. PAs have long seen their role as being a prominent voice for the profession to governments [4]. They promote their professions to the public, and support members when complaints are brought against them. They can often negotiate favourable professional indemnity insurance rates for members, provide access to research libraries and databases, and organise continuing professional development (CPD) events. The advantages seem so obvious that the authors were perplexed as to why in Australia, about 1 in 3 chiropractors choose not to belong to either of the two PAs [5, 6]. This is not the same for other health professional organisations in Australia. For example, over 80% of physiotherapists and osteopaths are members of a professional association (PA) [7, 8].

Only two previous studies have examined determinants of professional chiropractic association membership, one in Wales [9] and the other in Australian chiropractic students [10]. These studies have identified both inhibitors and facilitators to joining a PA. For chiropractic students, the influential factors on deciding whether or not to join a PA related to developing the profession, developing personal practice, the reputation of the PA and intra-professional communication [10]. Dispositional factors, such as personal values and opportunities for networking were also important. The sample of registered chiropractors from Wales also identified these factors as well as access to professional indemnity insurance, positive attitude to research and cost of membership [9].

### Aims

This qualitative descriptive study had two objectives: 1) to explore the views of non-member chiropractors about PAs in general; and 2) seek to understand the motivations of non-member Australian chiropractors about not joining a PA.

## Methods

### Rationale

Given the lack of published Australian studies examining the influences on professional chiropractic association membership, and that one in three Australian chiropractors do not belong to a PA, it is important to undertake research that addresses this issue. To understand this, a qualitative approach focusing on identifying the meanings behind an individual’s choice not to belong to a professional chiropractic association was undertaken. Qualitative research has the ability to bring the honest narratives and shared experiences of people to the fore by providing detailed description of the topic under study, and, through the interpretation and insight of the researcher, can make sense and coherence of the data presented [11].

A qualitative methodology was chosen for several reasons. First, it was anticipated there would be difficulty in accessing sufficient numbers of non-PA members for a quantitative study as they do not belong to common database such as a PA. Also, there was a lack of any previous data to inform the construction of a quantitative approach such as a survey. Consequently, this methodology allowed access to the valuable personal insights from the actual people who are the owners of this experience, or in other words the ‘experts’ of this phenomenon [12].

### Ethics

Ethics approval was obtained from the University Human Research Ethics Committee (2019/167) before recruitment and data collection. The study followed the COREQ guidelines for qualitative studies [13].

### Participant recruitment

Inclusion criteria included full-time or part-time practicing chiropractors in Australia who had been a self-reported non-member of any chiropractic PA for 3 years or more.

A purposeful sample of chiropractors, that is, those who were not members of a chiropractic association, were sourced with the number of participants for the study not determined. It was hoped that enough participants would be found, and that data saturation would be reached. Data saturation occurs when gathering fresh data no longer elicits new responses or information [12]. It was anticipated that between 6 and 15 participants in total may be required [12].

Potential participants were recruited by placing a request in an Australian Chiropractic Facebook Group (881 members). This on-line group was thought by the authors to be easily accessible and highly likely to contain practitioners who were non-PA members. In addition, snowball sampling was also used which involved participants and identifying additional participants from among their professional acquaintances. This sampling is a non-probability sampling technique used by researchers to identify potential participants for studies where respondents are hard to locate [14].

The first 8–12 chiropractors to respond to the invitation notice were emailed an information letter (Additional File 1) that detailed the purpose of the study, the research team, what participation entailed and their rights as participants. If they were agreeable to this, then they were invited to respond by signing the written consent and returning it to the lead investigator who enrolled them into the study. A suitable interview time for a Skype / Zoom / telephone interview was then arranged. Informed consent was obtained from each participant before commencing the interview. Repeat interviews were not carried out.

### Data collection

This was a qualitative descriptive study utilising in-depth semi-structured interviews with open-ended questions, at a time suitable to the participant via Skype / Zoom and telephone due to COVID-19 restrictions on face-to-face interviews. The interview questions were adapted from recent research that identified a number of barriers and facilitators to students considering chiropractic professional membership [9, 10]. The full interview script (*aide de memoir*) is included in Additional file 1.

The principal researcher (SI) conducted the interviews (*n* = 9). The nine participants (2 Facebook, 7 snowball sampling) were provided with the information sheet prior to the interview and invited to reflect on factors that might influence chiropractors to decide not to join a PA. Participants were also invited to make further comments as they felt appropriate to the topics under discussion during the interview. Participant responses were audio recorded on two digital devices and transcribed verbatim. The principal researcher also made notes to highlight important points and key aspects as they emerged at the time of the interview. Verbal consent was obtained prior to commencing the interview.

### Data analysis

All interviews were imported and analysed using the qualitative analysis NVivo 12 software [15] in conjunction with manual coding and thematic analysis as outlined by Braun and Clarke [16], who note that repeated readings results in familiarisation of the data and leads to identification of recurrent patterns and themes.

Trustworthiness of data and interpretation of the study involved four categories: credibility, transferability, dependability and confirmability [17]. To increase credibility, the transcriptions were returned to the interviewees for verification of accuracy. This ensured verification of data. The interviewer was familiar with the relevant literature and this helped ensure credible interpretation of the interactions with the participants, thus improving methodological rigour [18]. To attain dependability and confirmability of the data, the analysis process was reviewed by another qualitative expert (VC). The thematic analysis was supported by verbatim excerpts from the transcribed interviews and by reviewing field notes and reflective memos made during the interviews. Memos, field notes and reflections are a source of qualitative data as qualitative researchers aim to present a holistic account or the larger picture of the topic of interest [11]. Following these steps, the researchers were able to identify key concepts surrounding non-membership of chiropractic PAs.

Interviews were conducted, transcribed, coded and analysed in-turn. The transcripts were reviewed by the lead researcher and then further reviewed and discussed with a qualitative research investigator (VC), as to whether thematic saturation had been reached. The researchers agreed that saturation was achieved after the ninth interview. Consequently, no further participants were sought.

## Results

### Participants

The interviews were conducted by an experienced interviewer (SI) from January to April of 2020 and lasted an average of 30.2 min (25–42 min). There were nine participants (6 male, 3 female) with an average age of 38.8 years (24–57). Two described themselves as practicing in a rural setting, with a mean 11.3 years of practice (3–34) and 9.9 years of non-membership (3–31). Other characteristics of the sample are not given to protect the anonymity of participants.

### Findings / recurring themes

Five themes were identified from the data. They were: “Tarnished image of the profession”, “Not worth the money”, “Going it alone / What’s in it for me?”, “Two warring factions”, and “Lack of visibility”.

#### Theme 1: “Tarnished image of the profession”

The most frequently recurring theme mentioned by all respondents (a total of 29 references) was a perception that the profession of chiropractic has a poor standing in the eyes of the public and other health professionals.

R1 “we worked so hard to be chiropractors. And we study so much and know so much about the human body in this area or domain. And we are looked upon as worthless by the greater community. I don't like that. And it's not fair. . . it's the only profession that has this sort of thing happening to it that's going through a university-based model”.

Respondents thought the profession lacked homogeneity, unity, that it was without recommended guidelines of care, nor uniform treatments.

R8 “I'm not real comfortable with some of those other (chiropractic) techniques. And if they're big parts of gatherings I don't know whether I want to be part of that”.

This perception of a profession with a tarnished image appeared to reflect a low level of desire to engage with others via membership in a PA.

#### Theme 2: “Not worth the money”

All but one of the respondents viewed chiropractic PAs as lacking in value for money and not worth the cost of annual membership (18 references in total). Some mentioned that PAs offered access to reduced professional indemnity and malpractice insurance rates as a temptation to join but as not being sufficient. Others spoke about financial hardship and choosing not to join as a way of making savings.

R7: “My thoughts & the ones from the people that I've spoken to from what I've understood, number one is cost, they really don't see any value in it”.

#### Theme 3: “Going it alone/ what’s in it for me?”

Seven of the nine respondents expressed the view that chiropractic PAs were not thought of as providing anything deemed to be essential for practice or of direct benefit to themselves (21 references). It was mentioned by three respondents that the decision not to re-join the PA was made after forgetting or overlooking the renewal date as becoming aware that there was no discernible difference whether they belonged or not.

There appeared to be a high valuing of the individual and a low valuing of supporting the profession as a whole. In other words, self-referential thinking was central to the evaluation of the worth of PA membership.

R1 “I kind of plod along without it being any direct impact on me …. I can kind of get by with not having to be a part of them”.

Some spoke about being confident in their abilities and not needing the support offered by a PA, while others expressed discomfort with professional gatherings and assigned minimal value to group membership.

R4 “I haven't been part of that sort of group that get all funny about being professional and they go to all the seminars and all this sort of hype around the profession. I'm just not into it at all. Number one I don't have time to do it. And number two I just don't identify with that sort of professional thing”.

#### Theme 4: “Two warring factions”

Within Australia there are two PAs. One is thought of as providing open membership that embraces all chiropractors’ beliefs (from vitalism through to evidence-based practice) with the other describing itself as “evidence-based”. All but two of the interviewees expressed views that recognised this spectrum within Australia PAs but also more broadly within the profession and they did not want to be seen as part of this disunity (15 references).

R9 “The disagreement we have is obvious . . .some of us are on the philosophy or you know a lot of chiropractors do things that are unfounded, let's say, or unproven, or unresearched or whatever term they use, but there are some of us who are evidence based. So, there's always this form of dissociation from part of the profession and in itself shows that the whole profession is weak”.

#### Theme 5: “lack of visibility “

Six of the respondents expressed views indicating they perceived PAs as lacking a visible presence and this was perceived by some as being poor communication (15 references). Consequently, chiropractors were thought to not understand the full range of benefits that PAs provided. Respondents wanted this information to be easily accessible and were dissuaded from searching at the prospect of the level of difficulty involved.

R4 “So maybe there needs to be a bit more awareness about what they're actually offering and why they're even there in the first place. . . . it feels like sometimes there's all this information out there that you have to sift through and find it in some random website somewhere”.

This PAs were expected to be consistently working at being a high-profile presence in the day-to-day workplace with a range of relevant resources.

R6 “In general I guess making it more obvious, the benefits of joining. I mean I know that, and you know that on their websites, and they do have it …. to be honest you know, I kind of forget that they're there and unless I went and sought out that information”

## Discussion

### Overview

Although there are few studies that have examined factors that positively impact on non-chiropractic health professionals’ association membership, the most important predictor of membership seems to be an individual’s perception of the value of association membership [19]. In particular, an individual’s decision to join or remain in a PA depends on whether they perceive that the benefits outweigh the costs [19]. We will consider each of the five themes that emerged from our research individually and propose solutions.

#### Theme 1: tarnished image of the profession

The experiences of interviewees provided insights into a chiropractic profession that they thought to be internally conflicted and viewed negatively by the wider community and other health professionals. This view appeared to cast a shadow over all the respondents’ deliberations. The respondents’ views on the respectability of chiropractic are not without reason. The occupation of chiropractic is not rated as prestigious by the public [20] nor by other healthcare professions [21]. This is not helped by an inadequately informed public about the nature of chiropractic care in Australia [22, 23], and not unique to the profession of chiropractic. Studies with other health care professions have shown that scandals in PAs [24] and disagreement with PA positions and policies [2] resulted in lower levels of membership. Finally, it should be borne in mind, that a person’s self-esteem is influenced by the degree to which other people value their role, and this may also be a contributing factor [25].

There is a body of research exploring image repair that is relevant for Australian chiropractic PAs [26]. The main strategies are thought to be denial, evading responsibility, reducing offensiveness, mortification and corrective action [27]. When applied to chiropractic PAs, denial may involve convincing non-members that PAs did not create the current image for chiropractic, whereas evading responsibility could be to claim that the blame lay elsewhere e.g., a recalcitrant minority. Reducing offensiveness may be seeking to minimize the importance of the respectability issue and turn the tables by attacking those who raise accusations while at the same time promoting the PAs positive aspects. PAs could also communicate regret (mortification) and engage in corrective actions aimed at rectifying the damages. Finally, they could consider inoculating their members against likely future attacks against the profession by forewarning them of pending likely reputational attacks as well as providing members with counter arguments that weaken this threat. All of this presupposes that PAs have the resources (financial, expertise, person-power) to undertake these tasks and this may not be the case. It also assumes that image is the only barrier for non-PA members and this study suggests this is not the case.

Interestingly Australian chiropractic students, whose membership is free to PAs, did not see the image of the profession or PA as a barrier or facilitator to membership [10]. Perhaps this may be explained by the student population not having been exposed to the financial reality of day-to-day practice and persistent negative community opinions of chiropractic.

#### Theme 2: not worth the money

The views of the respondents in this study were reflected in the findings of previous studies of the importance of benefit outweighing cost as an influencing factor for PA membership [19]. Perhaps this can be explained by Rational Choice Theory which posits that individuals are purposive and intentional, seeking to achieve their given preferences [28]. To this end people tend to make choices to maximize their chances for achieving their preferences, generally driven by some tangible benefits [29]. Factors that were viewed as costs in this study and common to other health professionals were excessive membership fees [2], PA scandals [24] and disagreement with their positions and policies [2]. This study did not resonate with other health professions that PA involvement impacts negatively on their work life balance [30]. Shared factors that were viewed as benefits by the interviewed chiropractors in this study, Australian chiropractic students, and Welsh chiropractors were well priced membership while promoting the public and professional image of chiropractic, promoting research, providing workplace support and access to continuing professional development activities [9, 10]. Continuing education and networking has also been found to be important to other health professionals [19, 31].

Several respondents suggested that PAs could help chiropractors improve their practices by making clinical guidelines available. This function already occurs, as PAs provide seminars and continuing professional development to help members interpret and effectively use existing guidelines. Two possibilities exist. First the message of their availability is not reaching all members of the profession. Second, we have noticed that chiropractors seem to prefer ‘feel good’ seminars rather than research and evidence-oriented ones. This observation may warrant further investigation.

#### Theme 3: going it alone / What’s in it for me?

The narratives of many participants seem to echo aspects of the choice to belong to large groups, however their focus was on their own individual needs rather than on those of the wider group [32]. It is possible that this may reflect an increasing trend among younger health professionals where PAs are perceived as lacking in personal relevance [32].

Chiropractic practice in Australia can be insular, arising from a variety of historical, educational, geographic, and professional factors [33–35]. Therefore, the finding of an individualistic mentality should not be surprising. Historically, chiropractic developed outside mainstream medicine, and medicine viewed chiropractic as a competitor, driving chiropractors to practice on their own [36]. Some chiropractors also had and have an alternative paradigm of health and disease, leading them to have little or no association with medicine [37]. Chiropractic education in Australia, although predominantly within the public university system, tends to stand alone within those universities, rather than be integrated with other health care disciplines. Nearly all chiropractors in Australia work in private practice [6]. Just over 75% work with another practitioner and this is overwhelmingly either another chiropractor or a masseur [6]. Just over one-half of all chiropractors report receiving or making referrals to a medical practitioner and only 25% practice in more than one location [6]. Chiropractic is also largely excluded from the single-payer reimbursement system in Australia, and few chiropractors work in medical offices or hospitals [6].

Another factor thought to be influential, but not reflected in non-chiropractic PA members responses in this study, is the presence of broader social forces [3]. Here people organize themselves in order to assert their personal interests, enhance their personal reputation, and gain access to desired goods. The non-influence could be explained, at least in part, by the preference of ‘going it alone’. Other social motives are thought to influential were the seeking to belong for principles of solidarity or ideological (political, ethical, or religious) convictions, as well as to be emotionally associated with the community. These were echoed in the recorded interviews as unfulfilled desires and deemed to be an unlikely occurrence in PAs.

Recent research has looked at the degree to which people in a society are integrated into groups and this theoretical framework is known as ‘Individualism and Collectivism’ [38]. These two traits exist on a continuum, where those at the collectivistic end are more likely to show attentiveness to others and see themselves as exemplars of the larger group. At the individualistic end are people who show self-directedness, autonomy, and independence and see themselves as makers of their own destiny. On the surface it appears that chiropractic PA non-members are more likely to possess individualistic traits which warrants further investigation.

#### Theme 4: two warring factions

The chiropractic profession is not homogeneous. Some practitioners see the practice of chiropractic as being an evidenced-based approach to musculoskeletal care (MSK) of the spine [39]. Others have adopted an alternative paradigm and believe that spinal manipulation has a role to play in disease and wellness more generally (Non-MSK) by the removal of biomechanical lesions called subluxations that impact on the nervous system [40–42]. For example one Australian PA (A.C.A.) sees chiropractic as a health profession concerned with mechanical disorders of the musculoskeletal system and the effects of these on the function of the nervous system and general health but provides no website information or policy on subluxations [43]. The other PA (C.A.) sees it role as promoting evidence-based chiropractic care and inter-professional cooperation in order to improve community health through high quality, patient-focused care and has policy statements on subluxation as a historical concept, not supported by any clinical research evidence [44]. It is generally thought that the MSK and non-MSK factions exist as polar extremes with the majority falling somewhere in the middle [45]. The two factions strongly defend their own position and question the other’s paradigm [46–48], to the extent that a recent discussion paper proposed that these differences were irreconcilable and the profession should consider divorcing along these lines [46].

Several of the respondents in this study thought that PAs had a role to play by regulating substandard behaviour of its members. PAs are not empowered by statute to enforce laws or regulatory standards by suspending members from practice, or imposing fines. Their remedy is exclusion from the group and may not hold weight if the practitioner does not highly value membership in the first place. Perhaps they have the option of playing a role more broadly in addressing substandard behaviour by offering the public information about reporting such behaviours, publishing position statements on known issues, work alongside licensing / registration boards and advocate for the government to adopt positions that improve the standards of practice. The PAs potential to generate change is diluted when many in the profession have no interest in their particular definition of professionalism and will not join. This is further complicated when different chiropractors have fundamental differences in the definition of substandard behaviour. Ultimately this places PAs in a conundrum between needing as many members as possible to fund various activities and taking an ethical stand to represent the highest standards of professionalism. How PAs respond to this issue will most likely be influenced by their understanding of the reasons for their existence.

Finally, suggestions have been made that the way forward for the profession is through bringing these factions together and presenting a unified front by embracing all views to co-exist under the term chiropractor [49]. However, this ‘big tent’ approach, according to the respondents, may be another reason people do not to join, because they believe the association, in representing all permutations of chiropractic, does not adequately represent their particular views.

#### Theme 5: lack of visibility

The PAs were perceived to be of inconsequential value to the workplace and as struggling to be visible. It is easy to speculate, based on the findings of this study, that the lack of visibility is related to a lack of desirability. The participants’ views paint a picture of a squabbling profession with a poor image, whose PAs are thought to be costly and largely irrelevant and this fails to ‘catch the eye’ of the siloed Australian chiropractic practitioner.

Social media marketing may be one avenue for PAs to consider as this has demonstrated an ability to enable word of mouth as well as change attitudes towards a brand [50]. Most commonly this involves Facebook and Twitter, however other forms of social media such as virtual worlds, content community sites and sites dedicated to feedback have also been shown to enhance brand images [50]. The impact of marketing efforts in these domains are difficult to measure as the exact mechanism of how online sharing of information takes place and in turn shapes the co-creation of value by consumers remains largely unknown [50, 51]. Also, there are non-traditional marketing alternative strategies such as entrepreneurial marketing that better serve smaller organizations, such as PAs, and warrant consideration to overcome this proffered lack of visibility [52].

### Strengths and limitations

This is the first study to explore chiropractors’ views on why they chose not to belong to a representative PA. This study sampled the views of nine experienced Australian chiropractors with an average of nearly 10 years of non-membership in a PA. We are confident they have provided a rich insight into the issues surrounding non-membership in a chiropractic PA. However, as this was a qualitative study, our sample cannot be assumed to be representative of the views of all non-members of PAs nationally and internationally. However, the embedded messages inherent in the participants responses demand and direct attention to issues of concern for the profession. The authors are confident they have addressed the issues surrounding qualitative research of reflexivity [53], credibility, transferability, dependability and confirmability [18].

### Future research

Additional qualitative research could further explore some of the themes uncovered here. A quantitative survey of a representative sample of non-PA members will ultimately confirm the degree to which the factors identified by the small number of respondents in this study contribute to non-membership. Ideally this would involve chiropractic and non-chiropractic PAs from around the world to enhance its generalisability. On the surface it appears that chiropractic PA non-members are more likely to possess individualistic traits and any further studies should consider incorporating measures to this end.

This study when combined with the previous study of Australian chiropractic students’ attitudes to PAs suggests that, for some, there is a change for the negative in their view of the profession. An exploration of the factors surrounding this change would necessarily be longitudinal in nature.

## Conclusions

This qualitative study set out to examine the views of chiropractors who do not belong to a PA to gain insights into why this may be the case. The issues of non-PA membership are intertwined with the image of the profession more broadly. Namely, there is a perception of an unenticing profession that is internally conflicted and poorly rated by both the public and other health care professionals. PAs themselves are viewed as not providing enough support or resources on a day-to-day basis to justify the cost or the effort to search them out. There is the possibility that personal traits and societal pressures may act as modifiers or mediators. Non-members are looking for PAs to enhance the respectability of the profession in a manner that ultimately results in increased patient volume and the provision of readily accessible day-to-day resources and information. Many of these may be non-modifiable, and as such, present an enormous challenge for growing Australian PA membership. These results can inform the construction of a survey for the broader chiropractic non-membership community to confirm and expand upon these findings.

## Supplementary Information

**Additional file 1.**

## Data Availability

Yes, subject to obtaining additional Ethics approval.

## Abbreviations

CPD: Continuing Professional Development
PA: Professional Association
