# Supplementary material for: Factors influencing Australian chiropractors who choose not to join national professional associations: a qualitative study
Source: Chiropr Man Therap. 2020 Dec 1;28:58. doi: 10.1186/s12998-020-00351-4 (PMC7704117; doi:10.1186/s12998-020-00351-4)
Supplement: Supplementary file 1 — Additional file 1. [file 12998_2020_351_MOESM1_ESM.docx]

# Additional File 1.

## Information Letter
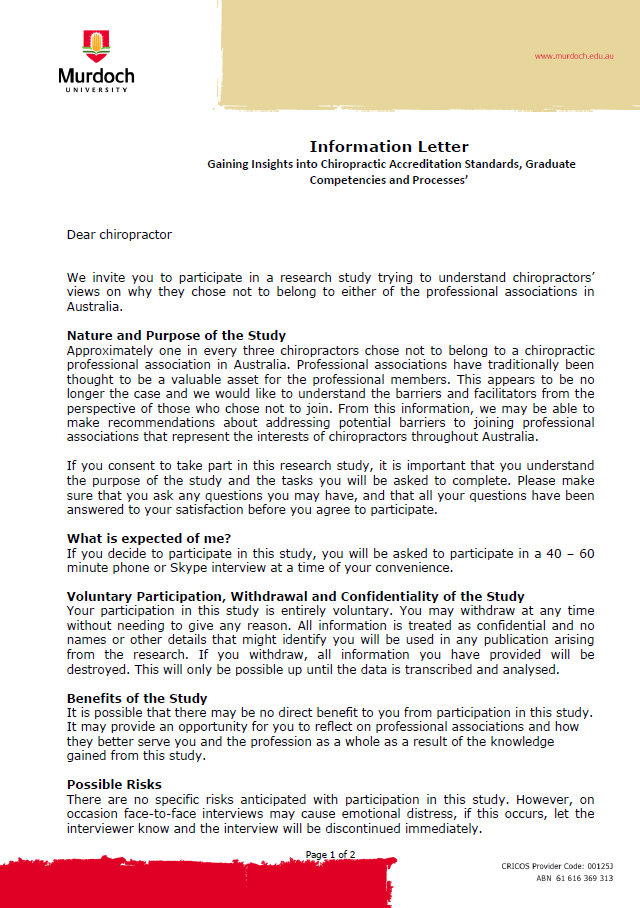


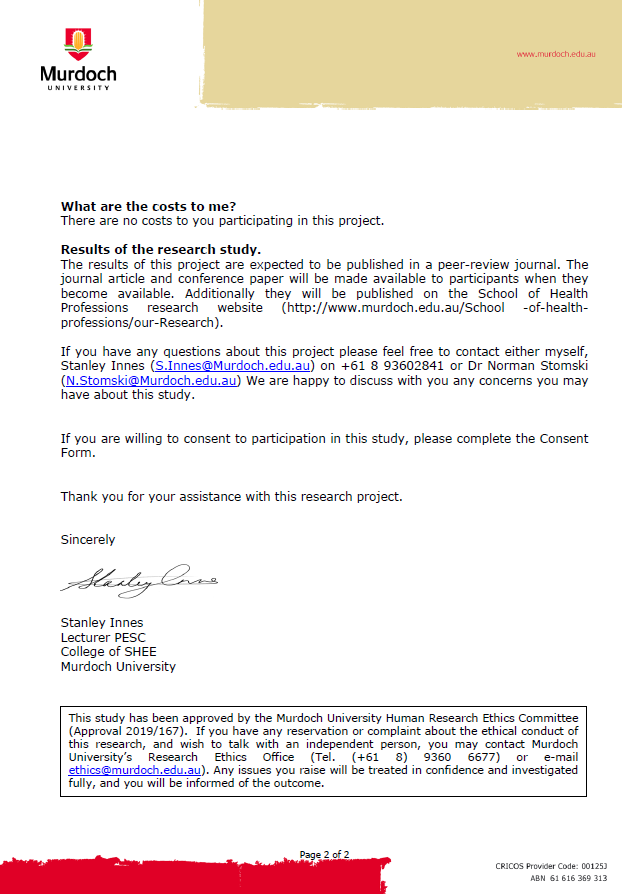


## Interview questions / *Aide de memoir*.

- How do you feel about the chiropractic profession?
- What are your views about chiropractic professional associations?
- What are your thoughts on why chiropractors might choose not to belong to a chiropractic profession association?
- What are your thoughts on things a chiropractic professional association could do to result in more members joining?

After the initial two open-ended questions, probing questions will be used to explore the responses further, these will include:

- How did you feel about...?
- How did you respond to...?
- Could you talk more about...?
- Can you explain that more?

At the conclusion of each interview the following questions will be used:

- How could chiropractic associations be improved to better meet the needs of chiropractors?
- That’s all I would like to ask, is there anything else you’d like to talk about or ask me?
